# AI-Driven Deep Learning Techniques in Protein Structure Prediction

**DOI:** 10.3390/ijms25158426

**Published:** 2024-08-01

**Authors:** Lingtao Chen, Qiaomu Li, Kazi Fahim Ahmad Nasif, Ying Xie, Bobin Deng, Shuteng Niu, Seyedamin Pouriyeh, Zhiyu Dai, Jiawei Chen, Chloe Yixin Xie

**Affiliations:** 1 College of Computing and Software Engineering, Kennesaw State University, Marietta, GA 30060, USA; lchen25@students.kennesaw.edu (L.C.); qli12@students.kennesaw.edu (Q.L.); knasif@students.kennesaw.edu (K.F.A.N.); yxie2@kennesaw.edu (Y.X.); bdeng2@kennesaw.edu (B.D.); spouriye@kennesaw.edu (S.P.); 2 Department of Computer Science, Bowling Green State University, Bowling Green, OH 43403, USA; sniu@bgsu.edu; 3 Division of Pulmonary and Critical Care Medicine, John T. Milliken Department of Medicine, Washington University School of Medicine in St. Louis, St. Louis, MO 63110, USA; zhiyudai@arizona.edu; 4 College of Computing, Data Science and Society, University of California, Berkeley, CA 94720, USA; jc01@berkeley.edu

**Keywords:** protein structure, computational methods, artificial intelligence, machine learning, deep learning, transformer, AlphaFold, protein modeling, bioinformatics, healthcare

## Abstract

Protein structure prediction is important for understanding their function and behavior. This review study presents a comprehensive review of the computational models used in predicting protein structure. It covers the progression from established protein modeling to state-of-the-art artificial intelligence (AI) frameworks. The paper will start with a brief introduction to protein structures, protein modeling, and AI. The section on established protein modeling will discuss homology modeling, ab initio modeling, and threading. The next section is deep learning-based models. It introduces some state-of-the-art AI models, such as AlphaFold (AlphaFold, AlphaFold2, AlphaFold3), RoseTTAFold, ProteinBERT, etc. This section also discusses how AI techniques have been integrated into established frameworks like Swiss-Model, Rosetta, and I-TASSER. The model performance is compared using the rankings of CASP14 (Critical Assessment of Structure Prediction) and CASP15. CASP16 is ongoing, and its results are not included in this review. Continuous Automated Model EvaluatiOn (CAMEO) complements the biennial CASP experiment. Template modeling score (TM-score), global distance test total score (GDT_TS), and Local Distance Difference Test (lDDT) score are discussed too. This paper then acknowledges the ongoing difficulties in predicting protein structure and emphasizes the necessity of additional searches like dynamic protein behavior, conformational changes, and protein–protein interactions. In the application section, this paper introduces some applications in various fields like drug design, industry, education, and novel protein development. In summary, this paper provides a comprehensive overview of the latest advancements in established protein modeling and deep learning-based models for protein structure predictions. It emphasizes the significant advancements achieved by AI and identifies potential areas for further investigation.

## 1. Introduction

Proteins are complex macromolecules that are important for the functions of living organisms [1]. They are made up of long chains of amino acids and linked together by peptide bonds. There are 20 common amino acids. These amino acids are composed of an alpha carbon, an amino group (NH_2_), a carboxyl group (COOH), a hydrogen atom, and a side chain. The side chain, also called the R-group, distinguishes one amino acid from another and determines its chemical behavior such as acidic, basic, polar, or nonpolar. Proteins are formed through a process called condensation reaction or dehydration synthesis. During this process, the carboxyl group of one amino acid interacts with the amino group of another amino acid. This interaction releases a molecule of water and forms a peptide bond. The created chain of amino acids is known as a polypeptide [1].

The protein structure can be classified into four levels: primary, secondary, tertiary, and quaternary structure. The primary structure is simply the sequence of amino acids in the polypeptide chain. The secondary structure is the backbone of the structure. They are usually alpha helices or beta sheets and formed by hydrogen bonds between the amino acids in the polypeptide chain. The tertiary structure is the protein’s overall three-dimensional (3D) shape. The interactions between the amino acid side chains determine it. The quaternary structure is the arrangement of multiple polypeptide chains in a protein [2].

Protein 3D structure prediction is the inference of the 3D structure of a protein from its amino acid sequence. Usually, the predicted structures are secondary and tertiary. There are several experimental methods for predicting protein structures including X-ray crystallography [3,4], nuclear magnetic resonance spectroscopy (NMR) [5,6,7,8,9,10], and electron cryomicroscopy (cryo-EM) [11]. Each technique has its advantages and limits [12,13,14,15,16,17]. For example, X-ray crystallography provides high-resolution structures and is well-suited for large proteins and complexes. However, it requires the protein to be crystallized, which can be challenging due to protein purity, crystallization efficiency, and crystal quality [18]. NMR measures 3D structures in their natural state and provides dynamics and intramolecular interactions, but it is limited to smaller proteins [13]. Cryo-EM preserves native structures, requires minimal amounts of samples for analysis, and does not need the protein to be crystallized. However, it requires a large protein complex, generally a minimum of 150 kDa, and certain homogeneity to achieve high-resolution determination [19].

Computational methods have been developed to predict the structure of proteins based on their amino acid sequence [20]. The accuracy of these methods has improved significantly in recent years [21]. All these experimental approaches demand time and resources. Computational methods complement those experimental techniques. The three main methods for protein structure prediction [20] are homology modeling [22], protein threading [23], and ab initio modeling [24]. Homology modeling uses a known protein structure as a template to predict the structure of a related protein. Protein threading predicts the structure of a protein by threading its amino acid sequence through a library of known protein structures. Ab initio modeling predicts the structure of a protein from scratch, without using any known protein structure as a template. With the introduction of artificial intelligence (AI) [25,26,27,28,29,30,31], machine learning algorithms increase the accuracy of protein structure prediction. Most of the machine learning algorithms for protein structure prediction focus on co-evolution-based methods. Co-evolution based methods use the evolutionary information contained in protein sequences to predict protein structures. Deep learning algorithms have also been utilized to extract intricate features from protein sequence data without making any intuitions. Accurately predicted protein structures can be used for drug discovery, antibody designs, understanding protein–protein interactions, and interactions with other molecules [21].

**Figure 1:**
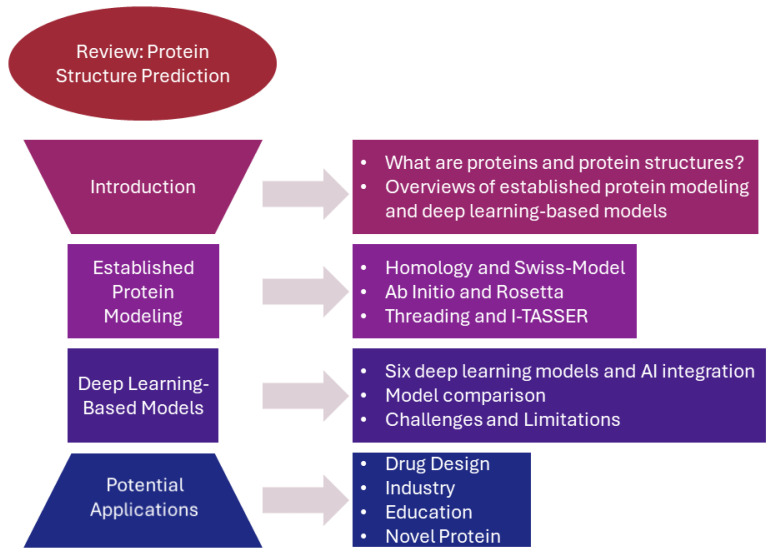
The flowchart of this review paper. It shows the overall flow of this paper, including the sequence of sections and their interconnections.

In summary, this introduction provides a brief overview of proteins, their structure, and computational methods. With this framework established, the next sections will explore in detail the computational methods applied to protein structure prediction. In this review paper, a wide range of computational methods used in protein structure prediction will be covered. After the established protein modeling section, it will not only provide some state-of-the-art deep learning models for protein structure predictions but also introduce how protein modeling integrates with AI. It will help computational biologists in this area to better understand the strengths and limitations of each approach and enable them to make reasonable decisions when selecting the most suitable method for a specific protein of interest. Figure 1 shows the overall flow of this review paper. It starts with an introduction to protein structure, followed by established protein modeling, deep learning-based models, and finally potential applications.

## 2. Established Protein Modeling

Before the introduction of AI, numerous computational methods were developed to tackle the protein structure prediction problem, based on different principles and assumptions. There are three types of protein modeling without AI: homology modeling, protein threading, and ab initio [20]. Table 1 shows the summary of all three types of protein modeling.

### 2.1. Homology Modeling

Homology modeling [22], also known as comparative modeling, predicts the structure of a target protein based on the experimentally determined structures of homologous proteins. It relies on the assumption that proteins with similar sequences have similar structures [20]. This method is the most accurate and widely used when suitable templates are available. Bayesian methods can be used to improve model accuracy by assessing the probability of different alignments. Swiss-Model [32] is an automated web-based platform that focuses on homology modeling, using the structural information of known proteins to help reveal the unseen folds of new sequences. Since its inception in 1993 [42], Swiss-Model has gone through continuous development and utilizes cutting-edge algorithms. It strives to produce high-quality structures [43,44]. The core strength of Swiss-Model sits in its highly automated workflow. By simply submitting a protein sequence, users can initiate the modeling process, with the platform identifying suitable templates from its enormous database, the SWISS-MODEL Template Library [43]. This library, regularly updated with newly solved structures, currently houses over 1 million entries (chains). This results in a high probability of finding relevant templates for a wide range of proteins [45]. In addition, rigorous model quality assessment ensures the trustworthiness of the results. It will provide users with metrics like QMEAN [46] to evaluate the predicted structure. Due to Swiss-Model’s free availability and user-friendly interface, it promotes scientific collaboration. Its integration with other bioinformatics resources, such as UniProt [47,48] and Protein Data Bank (PDB) [49], further enhances its utility. However, it is also important to notice the limitations of Swiss-Model. Primarily, the accuracy of the predicted structure is based on the availability of suitable templates. For unique proteins that lack close relatives in the database, the models might be less reliable. Also, the platform is less suited for handling exceptionally large or structurally complicated proteins alone.

### 2.2. Ab Initio Modeling

Ab initio modeling [24] is also known as de novo modeling [50], physics-based modeling [51], or free modeling [52]. It predicts the 3D structure of a protein solely from its primary amino acid sequence without any existing structural templates or homologous proteins. Ab initio methods explore different conformations that seek the native fold that corresponds to the protein’s functional state. Bayesian approaches can assist in sampling conformational spaces by integrating prior knowledge and uncertainty into predictions. Rosetta has been a software suite since 1999 [35]. It contains a wide range of modules and algorithms optimized for macromolecular modeling and protein folding. It uses complex energy functions that evaluate the stability and feasibility of protein conformations that guide the exploration of vast conformational spaces. Rosetta’s sampling algorithms explore the energy landscape efficiently and speed up the identification of low-energy protein structures [53]. Rosetta can not only perform ab initio folding, but also homology modeling. By harnessing sequence similarity between the target protein and its homologs, Rosetta aligns the target sequence with the templates and utilizes their structural information to construct a model of the target protein. There are many developed tools for sequence alignment and homology detection, including but not limited to HHSearch [54], Sparks [55], RaptorX [56], BLAST [57], PSI-BLAST [58], FFAS03 [59,60] and 3D-Jury [61]. This method is highly effective when suitable templates are available, allowing Rosetta to produce high-quality structural models even for proteins with limited sequence identity to known structures. Due to Rosetta’s comprehensive suite, it incorporates many techniques for protein structure prediction and design. Rosetta can be considered as a threading model, a topic that will be examined further in the subsequent paragraph. The integration of multiple modeling techniques within Rosetta’s framework not only enhances its predictive power but also enables researchers to explore the intricate relationship between sequence, structure, and function in proteins [62,63,64].

### 2.3. Threading

Threading [23] is also known as fold recognition [65]. Unlike homology modeling, threading goes beyond the limitations of sequence homology by integrating structural compatibility into the template selection process. It chooses suitable template structures from databases like the Protein Data Bank (PDB) [49]. The selection is based on both sequence similarity and the structural feasibility of aligning the target sequence onto the template backbone [66]. The key difference between threading and homology modeling lies in their approach to template selection and model generation. Homology modeling primarily relies on the assumption that proteins with similar sequences share similar structures and selects templates based solely on sequence similarity to the target protein. In contrast, threading considers both sequence and structural information to identify templates that not only have similar sequences but also exhibit compatible overall structures and spatial arrangements of secondary structure elements [67]. This structural alignment enables threading to predict the structure of proteins with limited sequence similarity to known structures, thus increasing the scope of protein structure prediction to include proteins with diverse sequences and folds. Bayesian methods can improve the accuracy of template alignments by probabilistically evaluating alternative alignments and refining model quality. Threading is the protein structure prediction method of choice when (1) the sequence has little or no primary sequence similarity to any sequence with a known structure, and (2) some models from the structure library represent the true fold of the sequence [68]. I-TASSER (Iterative Threading ASSEmbly Refinement) [37,38,39,40] stands as one of the major methods in the realm of protein structure prediction. Leveraging a hierarchical approach, it integrates threading, ab initio modeling, and structural refinement to generate accurate protein structure predictions [40]. One of its distinguishing features is the iterative refinement process, which iteratively improves the predicted models through both structural assembly and atomic-level refinement [39]. I-TASSER also aligns the selected templates to the target sequence, and the initial construction of the protein structure is guided [69,70]. After template-based modeling, ab initio modeling techniques are applied to generate additional structural fragments for regions lacking significant threading templates. Through multiple cycles of assembly and refinement, the initially generated models go through systematic improvements and gradually converge toward more reliable structures. This strategy makes I-TASSER address the inherent challenges, such as conformational sampling and energy optimization [39,71]. I-TASSER integrates diverse structural assessments and scoring functions to evaluate the quality of generated models. These assessments contain both global and local structural features and produce comprehensive evaluation and selection of the most plausible models. The incorporation of consensus scoring methods further enhances the reliability of the final predictions by integrating multiple scoring metrics [72]. The adaptability of I-TASSER extends beyond single-chain protein modeling, as it also supports the prediction of protein–protein interactions and protein–ligand binding modes. This broad applicability underscores its utility in various research domains, ranging from fundamental biological studies to drug discovery efforts [37]. Overall, I-TASSER represents a robust computational tool that continues to advance the understanding of protein structure and function through accurate and efficient prediction methodologies.

## 3. Deep Learning-Based Models

**Figure 2:**
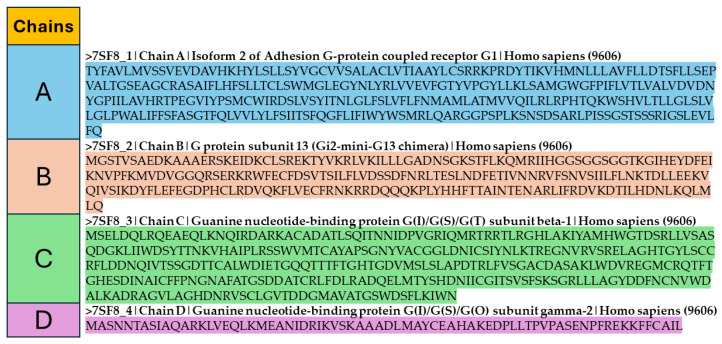
Sample FASTA file for protein (PDB ID 7SF8 [78]) with 4 chains.

In recent years, protein structure prediction has gone through outstanding breakthroughs, mostly driven by the integration of AI tools [21,73]. Researchers have developed advanced algorithms capable of inferring protein structures from limited information. In this section, some AI models’ fundamental principles are introduced and analyzed. Usually, the protein sequence, such as in Figure 2, is the primary source of inputs for a protein structure prediction using deep learning models. However, deep learning models essentially compute numbers. The raw protein sequences are represented by English alphabet letters. The deep learning models can not compute the letters. Various encoder methods can be used to derive the features from the protein sequence, such as One-hot encoding [74], Sequence-Statistics-Content [75], Position Specific Scoring Matrix (PSSM) [76], and K-Separated Bigram PSSM [77]. They all convert the amino acids into numbers or vectors of numbers. Other sources of information can also be used. For example, the physicochemical properties like hydrophobicity, polarity, charge, etc. Then, the outputs will be predicted, which contain 3D coordinates of atoms in the amino acids. The number of atoms being predicted depends on the specific problems. Predictions with more details require more atoms to be predicted.

**Figure 3:**
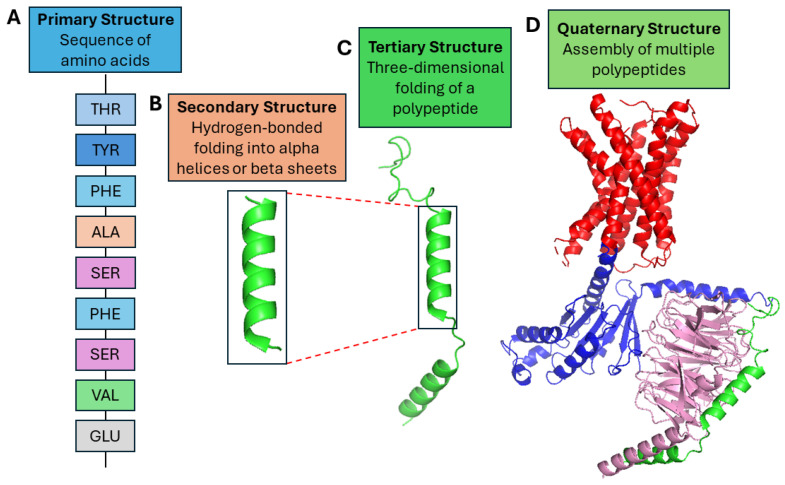
Four levels of protein structures. (**A**) The primary structure is shown as 3-letter codes, unlike Figure 2. The sequence is randomly written as a demonstration. (**B**–**D**) The secondary structure shows alpha helices as an example. Secondary, tertiary, and quaternary structures are visualized in PyMOL [80], a visualization tool for molecules, and macromolecules like proteins. The PDB ID used is 7SF8 [78].

FASTA [79] file format is commonly used as a method of storing biological sequence data, including DNA, RNA, and protein sequences. The standard data entry includes a header line that begins with the “>” symbol, followed by a distinct identification, a description (if applicable), and finally the actual sequence data. Due to their simplicity and versatility, FASTA files are widely used for the storage and exchange of sequence information. Figure 3 shows the protein structure levels. The primary structure is shown as 3-letter codes unlike Figure 2. Secondary, tertiary and quaternary structures will be the model outputs depending on the problem. The secondary structure shows alpha helices as an example. Secondary, tertiary, and quaternary structures are visualized in PyMOL [80], a visualization tool for molecules, and macromolecules like proteins. The PDB ID used is 7SF8 [78].

### 3.1. AlphaFold

AlphaFold [81], developed by DeepMind, is an AI system that revolutionizes protein structure prediction. AlphaFold integrates deep learning, mainly convolutional neural networks [82], with physical and biological knowledge about protein structures. It uses multi-sequence alignments (MSAs) to enhance its predictions. A newer version of AlphaFold, AlphaFold2 [25], is built on a neural network architecture known as a Transformer [83]. Transformers were introduced in 2017 and have since become a powerful tool across various fields because they can capture complicated relationships and patterns in entire sequences using attention mechanisms. This makes them suited for tasks like natural language processing. AlphaFold2 takes advantage of such an approach. In the CASP14 (Critical Assessment of Structure Prediction) competition, it outperformed other prediction methods by a significant margin. CASP is a biennial community experiment to determine the state-of-the-art methods in modeling protein structure. To accelerate scientific research, the AlphaFold database (DB) [84] provides over 200 million protein structure predictions. This database consists of a broad range of proteins, including the human proteome and those of 47 other key organisms. Researchers can freely access these predictions, contributing to advancements in bioinformatics and drug discovery. DeepMind and EMBL-EBI plan to continue updating the database with structures for newly found protein sequences. They also aim to enhance features depending on user feedback. The data are accessible under the Creative Commons Attribution 4.0 (CC-BY 4.0) license.

**Figure 4:**
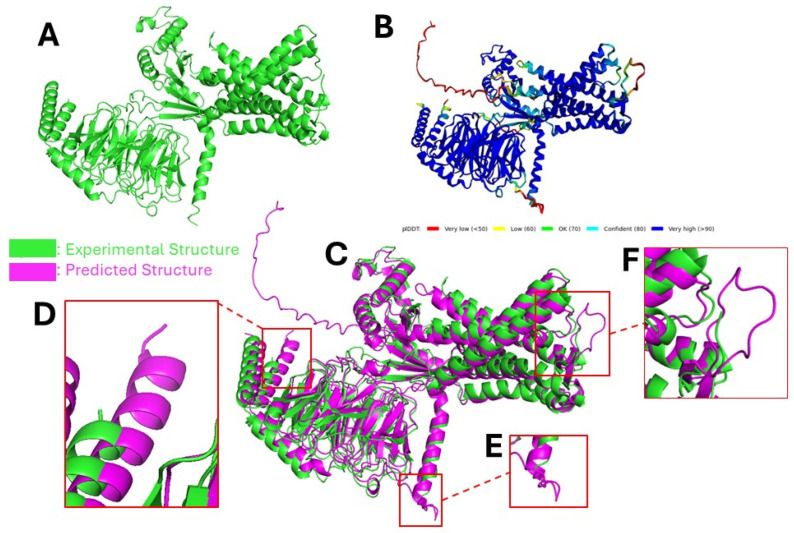
Comparison of experimental structure of protein (PDB ID 7SF8 [78]) and predicted structure by AlphaFold2. (**A**) PDB file of 7SF8 [78] shown in PyMOL [80]. (**B**) Predicted structure by AlphaFold2 with confidence scores using protein sequence (PDB ID 7SF8 [78]). A higher score means the model is more confident in the correctness of the predictions. (**C**) Figures (**A**) and (**B**) are shown together in PyMOL [80]. Purple is the AlphaFold2 prediction. (**D**–**F**) The zoomed-in area where AlphaFold2 has low confidence scores. There are some significant differences in these areas.

Figure 4 shows the AlphaFold2 prediction using protein sequence (PDB ID 7SF8 [78]) on Google Colab [85]. AlphaFold2 has overall high confidence in its prediction (see Figure 4B) for the majority of the structures. Most low-confidence locations are near the edge of the structure, where atoms are not stable (see Figure 4D–F). To some extent, AlphaFold2 also fills the gaps for some structures that cannot be determined by experiment.

The Protein Data Bank (PDB) [49] is a globally recognized repository that provides a standardized format for the storage of 3D structural information related to biological macromolecules, with a primary focus on proteins and nucleic acids. Each PDB file contains the atomic coordinates present in the molecule. It also includes supplementary metadata such as the experimental techniques used in structure determination, the authorship of the file, and the references cited. PDB entries also include information on secondary structure elements, ligand-binding sites, and crystallographic or NMR experimental details. The data in PDB files undergo rigorous quality control and validation processes. This makes the PDB files more accurate, reliable, and an essential resource for training and evaluating deep learning models in protein structure prediction.

AlphaFold3 [86] was released on 8 May 2024. Currently, it is only limited to non-commercial use through the AlphaFold server, unlike AlphaFold2 [25]. AlphaFold2 code is freely available on GitHub. A simplified version of it is available through the Google Colab notebook. AlphaFold3 can predict protein structures and interactions of all life’s molecules like proteins, DNA, RNA, ligands, and more because AlphaFold3 models the system as a collection of atoms. AlphaFold2 internally represents the protein structure by linking a rigid body frame with each amino acid relating to the alpha carbon atoms. The side chains were parameterized using χ-angles. This representation does not generalize arbitrary molecules. AlphaFold3 also groups atoms into tokens (residues for protein) for efficient computation and embeds the positions with a single matrix multiplication to allow limited spatial inductive bias. In AlphaFold2, Evoformer is applied over both MSA and residue pairs. In Alphafold3, it is replaced by a Pairformer that is applied over just the token pairs. As a result, it does not have column-wise attention like Evoformer. ReLU activation is also replaced by SwiGLU [87] in the model’s transition blocks. Another important difference is that AlphaFold3 uses a diffusion network for assembling its predictions. In AlphaFold2, the final structure was realized using invariant point attention. In AlphaFold3, it replaces this with a relatively standard non-equivariant point-cloud diffusion model over all atoms. According to the Google DeepMind AlphaFold team, for the interactions of proteins with other molecule types, AlphaFold3 sees at least a 50% improvement compared with existing prediction methods, and for some important categories of interaction, it has doubled prediction accuracy.

### 3.2. RoseTTAFold

RoseTTAFold [26,88,89], developed by Dr. David Baker’s team at the University of Washington, utilizes deep learning and evolutionary coupling to accurately and efficiently predict the 3D structures of proteins. RoseTTAFold combines the advantages of template-based modeling and ab initio modeling. Like AlphaFold2, RoseTTAFold uses MSA for predictions. The architecture exchanges information across the 1D amino acid sequence, the 2D distance map, and the 3D coordinates. The network analyzes relationships among sequences, distances, and coordinates. RoseTTAFold stands out for its capacity to quickly produce precise structure predictions. This makes it ideal for extensive proteome research and drug development projects. RoseTTAFold is also notable for its advanced prediction skills, as well as its easy-to-use interface and open-access design.

### 3.3. ProteinBERT

ProteinBERT [27] is a language model that utilizes transformers and has been designed specifically for protein sequences. It utilizes a self-attention mechanism inspired by BERT (Bidirectional Encoder Representations from Transformers) in natural language processing. It can acquire contextual representations of amino acid residues. The model has been trained using a comprehensive dataset consisting of around 106 million proteins obtained from UniRef90 [47,48]. It integrates global attention layers that replace traditional self-attention mechanisms. The linear complexity of these global attention layers allows the model to process protein sequences with different lengths without suffering the computational limitations that are typically quadratic complexity. ProteinBERT contains a novel task that specifically targets the prediction of Gene Ontology (GO) annotations. It gains a greater understanding of both local and global features within protein sequences by simultaneously learning to predict masked tokens and annotate proteins with GO terms. Researchers can extract embeddings at both the full sequence level and local (per location) level, using the innovative architectural aspects of this framework. This allows for a comprehensive representation that can be used for downstream tasks such as protein classification, function prediction, and structural analysis.

### 3.4. DeepFold

DeepFold [28] is a deep learning-based method for ab initio protein structure prediction. It uses the established energy calculations to guide a folding simulation. This method utilizes a vast number of predicted spatial constraints derived from powerful deep learning models. These precise constraints lead to a smooth energy landscape. It allows for efficient exploration and more accurate predictions compared to other cutting-edge methods. DeepFold operates in three key steps. DeepMSA2 generates diverse protein sequence alignments. DeepPotential predicts distances and interactions between amino acids. L-BFGS folding simulations utilize these restraints and the energy landscape for structure determination. DeepFold’s accuracy relies on the quality of the initial sequence alignments. There is great potential for further improvement by incorporating more advanced deep learning architectures and refining the method for generating MSA.

### 3.5. OmegaFold

OmegaFold [29] uses a pre-trained protein language model (OmegaPLM) for sequence modeling and a geometry-inspired transformer model (Geoformer) for structure prediction. By learning from a large collection of unaligned protein sequences, OmegaFold can predict structures without MSA. It offers advantages such as improved accuracy on orphan proteins and antibodies, and faster scaling compared to MSA-based methods. The method’s success can be attributed to the combination of OmegaPLM and Geoformer. OmegaPLM learns residue-level and pairwise embeddings, capturing structural and functional information. Geoformer makes these embeddings geometrically consistent. However, OmegaFold’s performance becomes worse when dealing with proteins that have very few sequence homologs compared to state-of-the-art methods like AlphaFold2. Despite this limitation, OmegaFold represents a significant step towards alignment-free high-resolution protein structure prediction. With further enhancements in model architecture and training strategies, its performance is expected to improve, particularly on targets with limited homologous sequences.

### 3.6. ESMFold

ESMFold [30,31], built upon the foundation of Meta AI’s ESM-2 language model, represents a big leap in protein structure prediction. ESMFold leverages ESM-2′s ability to capture protein sequence relationships to directly predict 3D structures from amino acid sequences alone. This bypasses the need for traditional methods that rely on extensive sequence homology, making it particularly advantageous for proteins with limited family data. ESMFold’s effectiveness also stems from its training on a vast protein sequence dataset, allowing it to model a wide range of protein structures with high accuracy. While ESMFold marks a notable improvement, it can sometimes falter with highly idiosyncratic proteins.

### 3.7. AI Integration

#### 3.7.1. Swiss-Model

Swiss-Model [42] integrates AI techniques to increase its capabilities. For example, it uses AlphaFold DB [84]. By searching this DB, Swiss-Model can find potential templates for protein structures that could be missed using traditional homology modeling techniques. This expands the pool of available templates and leads to more precise and trustworthy protein structure predictions. Swiss-Model’s AI integration also predicts quaternary protein structure [44]. This ability is essential for understanding protein function. Many proteins rely on interactions with other subunits to accomplish their biological activities. Swiss-Model uses machine learning techniques like Support Vector Machines (SVMs) [90] to assess the evolutionary constraints of protein interactions [32]. These constraints capture how much a certain region of a protein can vary in sequence compared to the rest of the protein surface. By examining interface conservation and the geometric properties of known protein complexes, Swiss-Model’s AI can identify templates that can have high-quality inter-chain contacts. This leads to a “quaternary structure quality estimate” (QSQE) score, which forecasts the expected reliability of interactions between subunits in the final model.

#### 3.7.2. Rosetta

Rosetta [35] also integrates AI to enhance its capabilities [91,92,93]. By using a wide range of experimentally determined protein structures, deep neural networks can identify patterns and features associated with native, biologically relevant conformations [94]. Subsequently, the trained models are integrated into Rosetta’s scoring functions. This results in a more precise assessment of the energy landscape and the detection of low-energy, stable structures [95]. AI-based methodologies, such as reinforcement learning and generative adversarial networks (GANs) [96,97], have been implemented to guide the sampling process. Through the process of acquiring knowledge from feedback signals and continuously improving the sampling strategy, these methods can explore the conformational space faster. As a result, they can reach native-like structures more quickly compared to traditional sampling strategies. A good example is trRosetta (transform-restrained Rosetta) [98]. It combines deep learning and Rosetta. The protein structure is constructed using direct energy minimization techniques and a constrained Rosetta. Deep neural networks will predict constraints such as inter-residue distances and orientation distributions.

#### 3.7.3. I-TASSER

I-TASSER [37,38,39,40] effectively incorporates multiple AI approaches across its hierarchical protein structure prediction process. The threading process discovers structural templates from the Protein Data Bank (PDB) [49] and depends largely on machine learning methods for sequence alignment and remote homology recognition. Advanced machine learning models are applied to identify subtle sequence–structure relationships, which enables I-TASSER to leverage even distantly related templates. In the fragment assembly stage, AI-based clustering algorithms group and organize the structural fragments excised from the identified templates. These algorithms examine the structural and sequence features of the fragments. This helps speed up the exploration of conformational space during model creation. Monte Carlo simulations are applied to develop candidate models by sampling the conformational landscape. These simulations are guided by sophisticated scoring functions that include physicochemical principles and structural insights acquired from machine learning models trained on known protein structures. The final refinement process also substantially integrates AI technologies. Machine learning-based scoring methods evaluate the quality of the generated models and guide the refining process toward more native-like conformations. AI-powered optimization tools, such as genetic algorithms and simulated annealing, intelligently navigate the complicated energy landscapes to select the most probable and stable models. This synergistic integration of AI approaches has been crucial in boosting I-TASSER’s accuracy and establishing it as a significant tool in the field of computational structural biology.

### 3.8. Table Summary

In Table 2, open source means that the code is available online, usually on GitHub. Google Colab [85] is a hosted Jupyter Notebook service that requires no setup to use and provides free access to computing resources. “Web-based” indicatesthat the creators have their websites to provide access to their models. AlphaFold2, ProteinBERT, OmegaFold, and ESMFold all utilize the transformer architecture. This suggests a trend towards the effectiveness of Transformer-based models in protein structure prediction. Even RoseTTAFold and DeepFold use the attention mechanism, which is a key component of the transformer model. The transformer was initially proposed for natural language processing. Its self-attention mechanism is what makes it stand out. It weighs the importance of different words in the sentence when processing information. This allows it to capture long-range dependencies in the data. In protein structure prediction, the protein sequences can be considered as a sequence of data, like words in a sentence. The transformer model can learn complex interactions between amino acids because of captured long-range dependencies. This leads to a more accurate prediction of protein structures. The models are all accessible through their websites or Google Colab. This makes them user-friendly and shows a trend in cloud-based computing for protein structure prediction tasks because it requires more computational resources to run deep learning models.

### 3.9. Model Comparsion

Experimental validation of AI models in protein structure prediction is crucial for assessing their accuracy. Recently, CASP model performance was judged overall by the global distance test total score (GDT_TS). It is a measure of similarity between two protein structures and is most commonly used to compare the results of protein structure prediction to the experimentally determined structure. Its values range from 0 to 100, where 100 means a perfect prediction. It calculates the percentage of C-alpha atoms in the predicted structure that are within a certain distance threshold of the corresponding atoms in the native structure. The final GDT-TS score is the average percentage across these distance thresholds. The original design calculates 20 GDT scores with 20 consecutive distance cutoffs (0.5 Å, 1.0 Å, 1.5 Å, … 10.0 Å) [99]. The cutoffs used in CASP are 1 Å, 2 Å, 4 Å, and 8 Å. Another metric often used in this area is called a template modeling score or TM-score [98,99], which is a measure of similarity between two protein structures. It ranges from 0 to 1, where 1 indicates a perfect match between two structures. It measures the distance between corresponding residues in the predicted and native structures, normalized by the length of the proteins. A TM-score is less sensitive to local errors and more focused on the overall topology.

During CASP14 in the year 2020, DeepMind’s AlphaFold2 [25] system demonstrated a major breakthrough. AlphaFold2 outperformed over 100 competing groups. AlphaFold2 achieved a median GDT_TS score of 92.4 in CASP14. The predictions were ranked by the sum of the Z-scores for all predictions. A Z-score is the GDT_TS score for one prediction minus the mean of all GDT_TS scores for the predicted target, divided by the standard deviation for all GDT_TS scores. For all 102 targets, AlphaFold2 achieved an average Z-score of 2.7506. The second best model, BAKER based on trRosetta [98], achieved 0.9910.

Google DeepMind did not participate in CASP15 in 2022. However, a lot of groups integrated AlphaFold2 into their models. A few groups showed a substantial improvement over the original AlphaFold2 [100]. But no group stood out like AlphaFold2 did in CASP14. Their Z-scores were very close. Yang-Server based on trRosetta received 1st place by a SUM Z-score of 90.4273. The second best model, UB-TBM based on I-TASSER [37,38,39,40], achieved 89.2119. However, this is with a penalty threshold of 0.0. A Z-score below the penalty threshold (either −2.0 or 0.0) will be assigned to the value of the threshold. When using the penalty threshold of −2.0, the 1st place is UB-TBM, with a SUM Z-score of 84.2212. The second best model becomes Yang-Server with 81.5826.

RoseTTAFold [26] is essentially an improved version of trRosetta. In fact, trRosetta is no longer available as a modeling option on the Robetta server, and users are encouraged to use the more accurate method, RoseTTAFold.

For 221 test proteins, DeepFold [28] receives average TM-scores of 0.751. RoseTTAFold and AlphaFold2 receives 0.812 and 0.903, respectively. It is expected because DeepFold was developed before the advances made by AlphaFold2.

OmegaFold [29] is the first model that successfully predicts high-resolution protein structure only based on sequence. It outperforms RoseTTAFold and achieves a similar prediction accuracy to AlphaFold2 on recently released structures. Unlike other models that require MSA, OmegaFold works without MSA, which means it runs faster and also works on divergent sequences (sequences without many homologs).

ESMFold [31] has a similar accuracy to AlphaFold2 and RoseTTAFold for sequences with low perplexity that are well understood by the language model. The power of ESMFold is that it runs significantly faster than other models. It can make predictions up to 60 times faster than a current state-of-the-art model like AlphaFold. Its speed and accuracy have made it possible to bridge the gap between the rapid growth of protein sequence databases and the slower development of protein structure and function databases.

Although CASP is a popular contest in the field of protein structure prediction, it is organized every two years. The results are not up to date after a certain time. For example, the latest CASP15 was hosted in 2022. The results of CASP16 will be available at the end of 2024. Continuous Automated Model EvaluatiOn (CAMEO) [101,102,103,104,105] complements the biennial CASP experiment. It conducts fully automated blind evaluations of three-dimensional protein prediction servers based on the weekly prerelease of sequences of those structures. It is a weekly contest. CAMEO also uses many performance metrics. For the final ranking, the default and main one is Local Distance Difference Test (lDDT) score [106]. It is similar to GDT_TS, and a superposition-free score that evaluates local distance differences in a model compared to a reference structure. It considers distances between all pairs of atoms in the reference structure lying at a distance closer than a predefined threshold. A distance is considered conserved in the evaluated model if it has the same length as in the reference within a tolerance threshold. The average lDDT score is calculated from thresholds of 0.5, 1, 2, and 4 Å. Local Distance Difference Test—Binding Site (lDDT-BS) is a variant of the lDDT scores. In short, this score is only calculated for targets where the experimental structure incorporates a ligand. A binding site is defined as the set of amino acid residues in the reference protein structure which have at least one atom within a 4.0 Å radius of any atom of the ligand.

From 7 July 2023, to 29 June 2024, OpenComplex [107] and Swiss-Model [32] are among the top two models for the submitted 711 targets. OpenComplex is based on AlphaFold2 [25] and OpenFold [108]. OpenFold is a trainable PyTorch [109] reproduction of AlphaFold2. For the default ranking results that use average lDDT, OpenComplex achieves 1st place with an average lDDT score of 81.7. The second best model is the Swiss-Model, with 79.2. For average lDDT-BS scores, the Swiss-Model is the highest, with 79. The second best model is OpenComplex, with 77.3. For all other metrics, those two models are still top two.

### 3.10. Challenges and Limitations

There are still many AI-driven models and architectures that are not mentioned in this article, like recurrent neural networks (RNNs) [110], graph-based convolutional networks (GCNs) [111], and long short-term memory (LSTM) [112]. Although these computational models with AI show great promise, there are still many challenges and limitations to be solved. The prediction of protein structure encounters many challenges and limitations that are inherent to its complex nature. These factors include computational complexity, effectively navigating the complex energy landscape, precisely sampling the conformational space, accommodating the flexibility of proteins, integrating models based on physics, ensuring accurate and reliable predictions, addressing concerns regarding limited data and data quality, and enhancing generalizability for a wide range of protein structures. In general, AI models, especially deep learning architectures, often require substantial computational power and resources. Training and fine-tuning these models can be resource-intensive and may not be accessible to all research teams. Many AI models, including deep neural networks, are often considered “black boxes” due to their complex nature. This lack of interpretability makes it challenging to understand how models arrive at specific predictions and to identify potential sources of error. Limited or biased datasets can lead to poor model performance and generalization. Although efforts are being made to improve data availability through collaboration and data sharing, inconsistencies and gaps in data still pose challenges.

A variety of inventive solutions has arisen in response to these issues. Distributed computing and advanced sampling techniques efficiently explore energy landscapes. Physics-based models combine machine learning frameworks, which capture complex interactions. Ensemble-based approaches and dynamic models handle protein flexibility. Collaboration improves data availability and quality. Researchers are exploring transfer learning and meta-learning strategies to improve generalizability across varied protein structures. This can construct more robust and versatile prediction models. Using interdisciplinary techniques and the exploration of computational and experimental procedures, these solutions make notable progress in the field of protein structure prediction.

AI-driven protein structure prediction also presents complex challenges in intellectual property, ethics, and data privacy. Determining ownership of AI-generated discoveries, such as novel protein structures and drug candidates, can lead to disputes over patent rights. Ethical concerns arise regarding equal access to AI-generated treatments. High costs may limit availability to disadvantaged populations. The protection of genetic and health data is crucial to prevent any misuse or illegal access. It requires robust privacy measures and clear consent protocols. Addressing these issues is essential for responsible and equal AI application in drug development.

## 4. Potential Applications

The utilization of AI in protein structure prediction has great potential in a wide range of fields [113], including the drug design industry [114,115], education [86], and novel protein [116,117,118,119]. AI-driven prediction models provide an understanding of the activities, relationships, and possible therapeutic targets of proteins by analyzing their 3D structures [25].

### 4.1. Drug Design

Researchers can obtain valuable knowledge about evolutionary relationships, functional divergence, and the underlying mechanisms that promote protein evolution [120] through the examination of structural similarities and differences among homologous proteins across many species. In the era of large-scale genomics and proteomics projects, protein structure prediction is important for annotating and characterizing the large amounts of proteins found by varying methods. Researchers can find binding sites and interactions that are important for drug targeting if they understand the spatial arraignment of proteins [121,122]. AI methods speed up this process by dramatically boosting the accuracy of protein structure prediction. By simulating protein–ligand interactions and predicting their binding affinities, AI also speeds up the development and optimization of new therapies [123]. By studying the 3D structure of target proteins, researchers can build and refine small molecule drugs or therapeutic antibodies that bind specifically to these targets [124]. This understanding promotes the development of more effective and selective drugs for treating diseases such as cancer, neurodegenerative disorders, and infectious diseases [125]. Personalized treatments can also be designed to target the unique molecular configurations of a patient’s proteins. These predictive models serve as an initiator for significant advancements in the fields of disease diagnosis, prognosis, and therapy [126] through the process of understanding the biological foundations of diseases and identifying potential targets for drug development.

### 4.2. Industry

Protein structure prediction has applications across many biotechnological and industrial businesses as well. For instance, in the food business, understanding the structures of enzymes and proteins can help improve food processing procedures, produce novel food additives, and raise the shelf life and quality of food products. Similarly, in the textile and paper industries, customized enzymes with specialized structures can be utilized to enhance manufacturing processes [114]. For a specific example, there are some companies that use AI to create novel therapeutics. Generate Biomedicines is a Boston-based startup that uses AI to design proteins. Its model is called Chroma [115]. This program uses a type of generative AI known as a diffusion model to design new types of proteins that have not been seen in nature. The diffusion model for image generations starts with a noisy image and gradually removes noise until a clear picture is formed. This can be changed to guide protein generation. On the Generate Biomedicines website, it shows their pipeline which includes programs in various stages of development across multiple therapeutic areas. The current targets include TSLP, TL1A, IL-13, SARS-CoV-2 S2 Domain, and SARS-CoV-2 RBD Domain. Another company called BenevolentAI [127] also uses AI to design proteins to treat diseases like Parkinson’s disease, heart failure, oncology, neurology, and immunology. These protein generators can be directed to produce designs for proteins with specific properties, such as shape, size, or function. This makes it possible to come up with new proteins to do particular jobs on demand.

### 4.3. Education

Tools like the AlphaFold3 [86] server can enhance the biology and bioinformatics curriculum. Students can engage with state-of-the-art technology and gain hands-on experience in protein structure prediction. This practical experience can not only reinforce theoretical knowledge but also prepare students for careers in research and industry. Instructors can use the server to demonstrate real-time protein folding and prediction. This makes abstract concepts more tangible. The use of AI in protein structure prediction encourages an interdisciplinary approach to education. Students from various fields, such as computer science, mathematics, and chemistry, can collaborate on projects involving AlphaFold3. This collaboration not only broadens their knowledge but also promotes a comprehensive understanding of how different disciplines intersect in the realm of biological research.

### 4.4. Novel Protein

The ability to predict protein structures also promotes the discovery and development of novel proteins with specific functionality [92]. Researchers can build synthetic proteins with desired properties like novel catalytic activity [116]. This capability opens opportunities for the invention of revolutionary biocatalysts, biosensors, and medicines [117,118]. Ai-driven protein engineering can speed up the production of bio-based products with applications from biodegradable polymers to renewable energy technologies [119]. Researchers can also understand mechanisms underlying cellular processes and disease states by accurately modeling protein–protein and protein–ligand interactions.

### 4.5. Future Research

As AI continues to evolve, it can provide opportunities for more innovation and discovery. Future research attempts may focus on developing prediction models to account for dynamic protein behavior, such as conformational changes and protein–protein interactions. The application of AI-driven protein structure prediction in domains such as agriculture, environmental science, and materials science holds the potential to address numerous global concerns, from food security to environmental sustainability [128]. For example, AI-driven protein structure prediction can impact our understanding of cellular energetic metabolism and biomolecular interactions. By using protein efficiency and allocation, these models provide information about phenotypic control mechanisms [129]. Furthermore, AI’s ability to analyze protein interactions at the nanoscale can lead to breakthroughs in both fundamental research and practical applications [130].

## 5. Summary

Proteins consist of amino acid chains that create primary structures with unique side chains controlling their behavior. The 3D structures of proteins involve primary, secondary, tertiary, and quaternary structures. Experimental methods including X-ray crystallography [3,4], NMR [5,6,7,8,9,10], and cryo-EM [11] have been used to determine the 3D structures. However, they all demand labor and resources. Computational methods have been developed as useful tools. They apply algorithms to predict structures based on amino acid sequences. These methods, including homology modeling [22], protein threading [23], and ab initio modeling [24], are increasingly accurate, often complementing experimental procedures. Machine learning techniques, particularly co-evolution and deep learning-based, further increase prediction accuracy.

Before the advent of AI, computational methods for protein structure prediction were developed to study protein function, interactions, and evolution. These methods include homology modeling, protein threading, and ab initio modeling. Swiss-Model [32], a widely used tool, automates the homology procedure based on sequence similarity to build feasible 3D models. Despite its dependency on suitable templates, Swiss-Model’s ongoing improvement and integration with other resources boost its usability. Rosetta [35] leverages physics-based algorithms to explore conformational spaces rapidly. It provides high-quality models even for proteins with minimal sequence identity to known structures. I-TASSER [37,38,39,40] integrates threading, ab initio modeling, and iterative refining to provide reliable predictions. Its hierarchical structure and consensus scoring boost reliability and make it relevant in numerous study disciplines.

AI has advanced the field of protein structure prediction. Models like AlphaFold2 [25], RoseTTAFold [26], and ProteinBERT [27] utilize AI to reliably predict protein structures from amino acid sequences. AlphaFold2 achieves outstanding accuracy as demonstrated in contests like CASP14. It is also one of the very first models whose predicted structures are practical. RoseTTAFold combines deep learning and evolutionary coupling, and ProteinBERT uses transformers and global-attention layers for pattern recognition. DeepFold was developed before the advances made by AlphaFold2, and it still shows decent performance. OmegaFold accuracy is similar to AlphaFold2 and works without MSA. ESMFold accuracy is also similar to AlphaFold2 and it runs much faster—up to 60 times. Existing approaches such as Swiss-Model, Rosetta, and I-TASSER have integrated AI to boost their predictive capabilities. AlphaFold2, trRosetta, RoseTTAFold, and I-TASSER models have shown top performance on CASP14 and CASP15. OpenComplex [107], which is based on AlphaFold2, and Swiss-Model showed the top performance on CAMEO from 7 July 2023, to 29 June 2024 with 711 submitted targets. The integration of AI into protein structure prediction has accelerated progress in understanding protein folding, function, and interactions. These AI-driven models [25,26,27,28,29,30,31] advance varied scientific fields, from biochemistry to drug discovery.

In drug design [122], accurate protein structure prediction improves the creation of targeted medicines, including the treatment of cancer and neurodegenerative disorders [125]. AI-driven protein engineering advances the development of novel proteins with specific functions, enhancing biocatalysis and biosensors [92,117,118]. The AI-powered prediction toos, like AlphaFold3 [86] server, increase teaching in molecular biology and bioinformatics, delivering engaging learning experiences and deeper knowledge [131]. In evolutionary studies, protein structure prediction helps in understanding functional divergence and evolutionary relationships [120]. In industries like food processing and textiles, protein structural insights improve manufacturing processes and product quality [114]. A Boston-based startup called Generate Biomedicines already leverages AI to design proteins. Further research may focus on dynamic protein behavior prediction and merging AI with experimental approaches for quicker structure determination. AI models’ interpretability can also be improved. This helps a better understanding of the principles that govern protein folding. AI-driven protein structure prediction could help solve global challenges in agricultural and environmental sustainability, displaying its potential for revolutionary influence.

In conclusion, the incorporation of AI into protein structure prediction not only helps our understanding of structural biology but also holds significant potential for tackling real-world difficulties in healthcare, industry, and education. Beyond its current capability, AI offers many advantages. For example, scalability allows AI to analyze large datasets and complex biological systems. With newly available data, AI can have a bigger database to infer the target protein sequence’s structures. As hardware and algorithms advance, AI will keep enhancing its capability of predictions. Continued research promises significant advances in biomedical research and beyond.

## Figures and Tables

**Table 1:** Summary of established protein modeling.

Method	Advantages	Disadvantages	Examples
Homology Modeling	1. Utilizes experimentally determined structures of homologous proteins. 2. Highly accurate when suitable templates are available. 3. Widely used and accessible.	1. Relies on the availability of suitable templates. 2. Less reliable for unique proteins lacking close relatives in the database. 3. Less effective for exceptionally large or structurally complicated proteins.	Swiss-Model [32], Modeller [33], Phyre2 [34]
Ab Initio Modeling	1. Predicts structure solely from amino acid sequence, no need for existing templates. 2. Effective in producing models for proteins with limited sequence identity to known structures. 3. Can explore vast conformational spaces to identify low-energy protein structures.	1. Computationally intensive. 2. Success depends on the accuracy of energy functions and sampling algorithms. 3. May struggle with proteins with novel folds or significant structural rearrangements.	Rosetta [35], QUARK [36], I-TASSER [37,38,39,40]
Threading	1. Considers both sequence and structural information for template selection. 2. Predicts structures for proteins with limited sequence similarity to known structures. 3. Increases the scope of prediction to include proteins with diverse sequences and folds.	1. Requires significant computational resources for template search and alignment. 2. Relies on the accuracy of threading algorithms and the structural compatibility of templates. 3. May produce inaccurate models if no suitable templates are found.	I-TASSER [37,38,39,40], HHpred [41], Phyre2 [34]

**Table 2:** Models’ environments and key architectures.

Model	Environment	Key Architecture
AlphaFold2 [25]	Open Source (Python), Cloud (Google Colab)	Transformer-based Deep Learning
RoseTTAFold [26]	Open Source (Python), Cloud (Google Colab)	Attention-based Neural Network
ProteinBERT [27]	Open Source (Python), Cloud (Google Colab)	Transformer-based Deep Learning
DeepFold [28]	Open Source (Python), Web-based	Custom Multi-Stage Deep Learning Pipeline
OmegaFold [29]	Open Source (Python), Cloud (Google Colab)	Transformer-based Deep Learning
ESMFold [30,31]	Web-based, Open Source (Python), Cloud (Google Colab)	Transformer-based Deep Learning
Swiss-Model [42]	Web-based	Homology
Rosetta [35]	Web-based	Ab Initio, Homology, and Threading
I-TASSER [37,38,39,40]	Web-based	Threading and Ab Initio
